# Interactions Between Anesthesia and Sleep: Optimizing Perioperative Care to Improve Sleep Quality and Surgical Recovery Outcomes

**DOI:** 10.7759/cureus.78505

**Published:** 2025-02-04

**Authors:** Sahil Patel, Raymond Ownby

**Affiliations:** 1 Osteopathic Medicine, Nova Southeastern University Dr. Kiran C. Patel College of Osteopathic Medicine, Davie, USA; 2 Psychiatry and Behavioral Sciences, Nova Southeastern University Dr. Kiran C. Patel College of Osteopathic Medicine, Davie, USA

**Keywords:** anesthesia, anesthetic agent, circadian rhythm, general anesthesia, sleep, sleep wake cycle

## Abstract

Sleep is a fundamental physiological process that supports immunity, cognition, memory, and metabolism, making it essential for overall health and well-being. Poor-quality sleep is associated with the onset of many diseases, including cardiovascular and mental health disorders. When using anesthesia, it is essential to have a thorough understanding of the metabolic dysfunction and immunosuppression linked to sleep loss to ensure appropriate perioperative management. Commonly used agents like propofol, sevoflurane, and ketamine affect consciousness, pain levels, and autonomic responses. Inhaled anesthetics (e.g., sevoflurane and isoflurane) and intravenous anesthetics (propofol) act on gamma-aminobutyric acid (GABA) receptors and influence stages of sleep and circadian rhythms. Ketamine may uniquely preserve some aspects of restorative sleep, while most anesthetics affect rapid eye movement (REM) and slow wave sleep (SWS), altering cognitive and physical recovery. Modifying anesthetic regimes depending on the patient's sleep history and risk factors can maximize sleep health and postoperative patient outcomes.

The physiological effects of anesthesia on the central nervous system persist beyond the perioperative period by influencing sleep quality, circadian regulation, and postoperative outcomes. Effective pain management is a significant component in addressing sleep quality. Opioids, while effective for pain relief, disrupt sleep architecture by reducing REM and SWS, increasing awakening frequency, and potentially causing respiratory depression. Multimodal pain therapy, including non-opioid analgesics, can reduce dependence, improve sleep quality, and lower adverse effects. Anesthetic agents can influence the body's internal clock, leading to mood changes, fatigue, and cognitive deficits. This review exploits the relationship between sleep and anesthesia, detailing the effect of anesthetic agents on the quality, architecture, and recovery of sleep patterns post-surgery. It also explores how these agents influence sleep stages, such as REM and non-REM sleep, and their implications for patient outcomes. Incorporating sleep optimization techniques can enhance recovery timelines and patient well-being. Researching anesthetic techniques that support postoperative sleep health is essential for further improving patient outcomes.

## Introduction and background

Sleep is a vital physiological process in humans and, if not acknowledged, can lead to the onset and progression of numerous diseases. During sleep, the body undergoes essential processes such as tissue repair, immune system strengthening, and the regulation of hormones [1]. Poor sleep patterns can impair pain tolerance and recovery from surgery; therefore, incorporating sleep health into clinical practice can improve chronic conditions' management and long-term care [2]. Appropriate anesthesia use is crucial in surgical settings as it influences patient outcomes, recovery times, and overall surgical success. Anesthesia can be categorized into three approaches: general anesthesia renders the patient completely unconscious, regional anesthesia blocks sensation in specific body areas, and local anesthesia numbs a localized area [3]. Anesthetic agents such as propofol, sevoflurane, and ketamine modulate consciousness, pain pathways, and autonomic nervous system responses [4]. Innovations in anesthetic techniques have significantly enhanced the safety and effectiveness of anesthesia by reducing the risk of complications and improving postoperative recovery [4].

A detailed understanding of the complex interplay between anesthesia and sleep is essential for improving patient care strategies. The primary aim of this review is to detail this relationship, specifically focusing on how various anesthetic agents can influence sleep quality, sleep patterns, and overall postoperative recovery. Surgical patients often experience sleep disruptions due to preoperative anxiety and postoperative pain, making the relationship between sleep and anesthesia a pivotal component of recovery. The effects of various anesthetic agents, including volatile anesthetics, intravenous agents like propofol, and adjunctive medications, on sleep stages such as REM and non-REM sleep will be explored [5]. By synthesizing existing literature, the discussion aims to highlight preoperative, intraoperative, and postoperative challenges while offering practical treatment recommendations for enhancing sleep throughout each stage of the surgical process.

## Review

Preoperative sleep disorders in surgical patients

According to the World Health Organization, 27% of the world population has a sleep concern, while 50% of surgical patients have preoperative sleep disturbances [6]. Sleep disorders can be categorized into several groups, including insomnia, sleep-related breathing disorders, central disorders of hypersomnolence (such as narcolepsy), circadian rhythm sleep-wake disorders, sleep-related movement disorders, parasomnias, and miscellaneous sleep disorders [7]. Studies indicate that preoperative sleep disorders can significantly affect the quality of postoperative awakening and pain levels, potentially due to heightened inflammatory responses in the body [6]. Additionally, the stress of undergoing surgery can intensify preoperative anxiety, further aggravating existing sleep disorders or triggering new ones. Preoperative anxiety is a prevalent condition, impacting up to 80% of surgical patients through direct activation of the autonomic nervous system and neuroendocrine alterations [8]. Preoperative anxiety can increase the anesthetic agent dose requirement, postoperative pain, infection risk, and delays awakening [8].

Obstructive sleep apnea (OSA) is a commonly diagnosed sleep disorder that is associated with significant comorbidities and perioperative complications [9]. Upper airway abnormalities characterize OSA, including a narrowed pharyngeal airway, enlarged tonsils, an elongated uvula, or excess soft tissue in the airway. [9]. Preoperative evaluation of OSA and implementing appropriate perioperative management and treatment are crucial in patient recovery. Patients diagnosed with OSA pose several surgical and anesthetic challenges, such as difficulty maintaining the airway, risk of hypoxemia, and complications related to the use of sedatives and opioids [9]. An obstructed airway will impose challenges in intubation and mask ventilation during surgery. Given the association between sleep and surgical outcomes, it is crucial to routinely assess sleep quality and implement strategies to mitigate sleep disorders before surgery. Conversely, patients with healthy sleep hygiene in the preoperative phase exhibit improved immune function and decreased surgical stress on the body [6]. Adequate and high-quality sleep improves preoperative psychological well-being, reduced anxiety levels, and greater patient satisfaction with the surgical experience.

Strategies for improving preoperative sleep

Improving sleep quality before surgery is essential for enhancing surgical outcomes, including shorter recovery times, reduced pain levels, and a lower risk of comorbidities. Various strategies have been identified to help patients enhance their sleep hygiene in preparation for surgery. Non-pharmacological methods are considered the first-line treatment for managing sleep concerns [10]. Simple techniques such as relaxation, breathing exercises, and listening to music have been shown to improve sleep in preoperative and postoperative settings [10]. If there is ample time and the sleep concerns are severe, cognitive behavioral therapy for insomnia (CBT-I) is considered the first-line intervention [11]. CBT-I, a highly effective method, is shown to produce results equivalent to sleep medications, with no side effects and fewer episodes of relapse [11]. The five components of CBT-I are sleep consolidation, stimulus control, cognitive restructuring, sleep hygiene, and relaxation techniques, which promote the body's natural sleep mechanism [11]. However, CBT-I intervention typically lasts six to eight weeks; therefore, screening for insomnia should be conducted well before the elective surgery [10]. Recent studies have shown that completing CBT-I results in the remission of insomnia and lower levels of C-reactive protein, interleukin-6, and tumor necrosis factor, indicating a decreased inflammatory response and improved healing [10]. 

Pharmacological treatment can also aid sleep problems; however, a conclusive preoperative risk assessment should be conducted to review the patient's current medication regimen, contraindications, and surgical associations. A well-studied conservative option is melatonin supplementation, which can improve sleep onset and duration in surgical patients by regulating circadian rhythms and sleep-wake cycles [10]. Exogenous melatonin can benefit the delayed sleep phase type of insomnia and circadian disorders [10]. Melatonin, known for its safety and minimal side effects compared to medications like benzodiazepines, can be used as an adjuvant therapy for anesthesia during the perioperative period [12]. Preoperative melatonin administration has numerous positive effects, including sedation, analgesia, anti-anxiety properties, reduced incidence of postoperative delirium, and improved sleep [13].

Studies have shown that preoperative patient education and counseling interventions have significantly improved outcomes and should become a prerequisite for elective surgery patients [14]. Counseling sessions are crucial in informing patients about practical sleep tactics, such as following a consistent sleep schedule, creating a comfortable sleeping space, and engaging in relaxation exercises [14]. Incorporating these instructional elements into standard preoperative care fosters a health-conscious approach and emphasizes the healthcare professional's role in recognizing the importance of sleep in surgical preparation.

Overview of anesthetic agents

Selecting the most appropriate anesthetic agents is essential, as it can profoundly affect postoperative recovery and the overall surgical experience. Different anesthetic agents impact pain management, consciousness, and the architecture of sleep, each influencing various aspects of recovery, such as the quality of awakening, the severity of postoperative pain, and the restoration of normal sleep patterns.

The mechanism of action of inhaled anesthetics is within the central nervous system through augmentation of chloride channels (gamma-aminobutyric acid (GABA) receptors) and potassium channels while depressing neurotransmission pathways [15]. Inhaled anesthetics are widely used in general anesthesia for their ability to facilitate rapid induction and recovery [16]. Anesthetic gases (nitrous oxide, halothane, isoflurane, desflurane, and sevoflurane), referred to as inhaled anesthetics, are used during the operative phase to keep patients unconscious and pain-free throughout the procedure and as adjuncts to intravenous anesthetic agents (e.g., midazolam and propofol) [16]. Compared to intravenous drugs, the quick therapeutic actions of these agents enable effective sedative induction and cessation, resulting in appropriate amnesia, anesthesia, and a faster recovery period after surgery [5]. For inhalational delivery, vaporizers are necessary for the volatile anesthetics (halothane, isoflurane, desflurane, and sevoflurane), which are liquids at room temperature [15]. All inhalational anesthetics cause immobility and amnesia, except for nitrous oxide, which also causes analgesia [15]. 

Propofol, an intravenous anesthetic, is widely used for its rapid onset and short duration of action [17]. It can be administered as a bolus, infusion, or a combination of both and is commonly utilized for procedural sedation, monitored anesthesia care, and general anesthesia induction [17]. Its mechanism of action occurs through GABA-mediated chloride channels in the brain [17]. Propofol increases the neurotransmitter's inhibitory effects by slowing down GABA's dissociation from GABA receptors in the brain [17]. It is commonly used for moderate sedation to general anesthesia, providing low organ toxicity and excellent compatibility with various other frequently used drugs in anesthesia [17].

Ketamine hydrochloride, commonly known as ketamine, stands out as a unique anesthetic. It can be used as a general anesthetic, either alone or in combination with other drugs. Ketamine inhibits hyperpolarization-activated cyclic nucleotide-gated potassium channel 1 receptor and is a noncompetitive antagonist of glutamate and N-methyl-D-aspartate (NMDA) receptors [18]. Its unique dissociative effects and partial agonism of opioid mu-receptors enable painful procedures to be conducted while maintaining a steady, comfortable, and sedated state [18]. When paired with other general anesthetic medications, ketamine can be used as a pre-anesthetic for the induction of anesthesia, particularly for short medical procedures that do not require skeletal muscle relaxation [18]. Unlike traditional anesthetics, ketamine induces a trance-like state, providing analgesia and sedation without complete loss of consciousness [18]. Recent studies have highlighted ketamine's potential to enhance certain aspects of the sleep-wake cycle, promoting deeper sleep and potentially mitigating postoperative pain [18]. Table 1 details the mechanism of action of the commonly used anesthetic agents.

**Table 1:** Classification of anesthetic agents. NMDA, N-methyl-D-aspartate; HCN1, hyperpolarization-activated cyclic nucleotide-gated potassium channel 1; GABA, gamma-aminobutyric acid

Anesthetic Agent	Mechanism of Action
Inhaled Anesthetics (Sevoflurane, Desflurane, and Isoflurane)	Increase signals to chloride channels (GABA receptors) and potassium channels while decreasing signals to excitatory pathways [15].
Intravenous Anesthetics (Propofol and Midazolam)	Increased the duration of GABA-activated channel opening, leading to increased chloride influx and hyperpolarization of postsynaptic neuronal membranes [17].
Local Anesthetics (Lidocaine)	Block voltage-gated sodium channels in nerve cell membranes, preventing action potential propagation and reducing pain sensation [3].
Ketamine	Inhibits HCN1 receptors and is a noncompetitive antagonist of glutamate and NMDA receptors, providing analgesia and sedation [18].

Anesthetic effects on intraoperative sleep architecture

**Figure 1:**
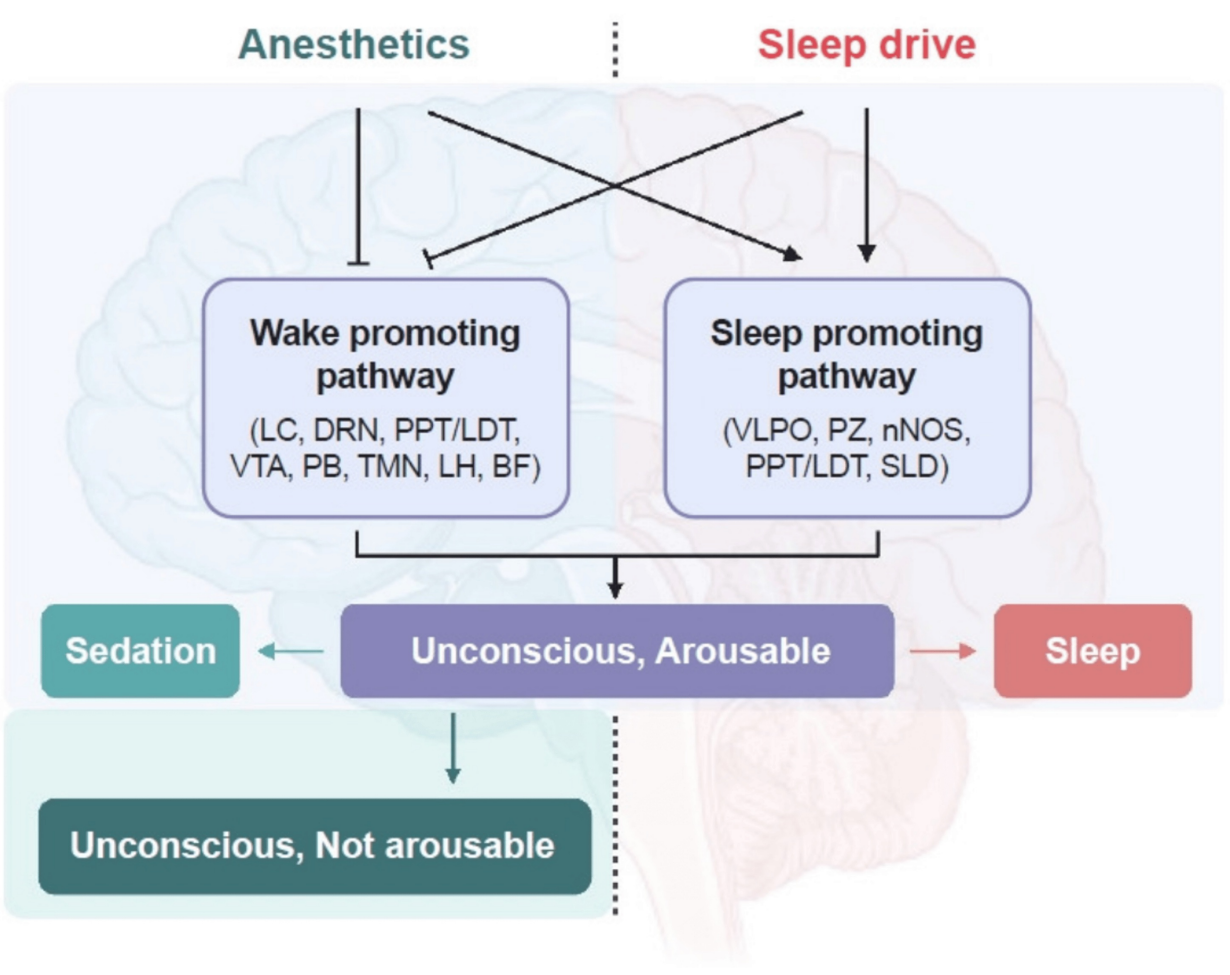
Similarities and differences in the mechanism of action of general anesthesia and sleep. LC: locus coeruleus, DRN: dorsal raphe nuclei, PPT: pedunculopontine tegmental nucleus, LDT: laterodorsal tegmental nucleus, VTA: ventral tegmental area, PB: parabrachial nucleus, TMN: tuberomammillary nuclei, LH: lateral hypothalamus, BF: basal forebrain, VLPO: ventrolateral preoptic area, PZ: parafacial zone, nNOS: neuronal nitric oxide synthase (nNOS)-containing neurons in the cortex, PPT: pedunculopontine tegmental nucleus, LDT: laterodorsal tegmental nucleus, SLD: sublaterodorsal nucleus. Copyright/license: This figure has been adapted from [20], which is an open-source article distributed under the terms and conditions of the Creative Commons Attribution Non-Commercial License (http://creativecommons.org/licenses/by-nc/4.0/)

General anesthesia and sleep share similar mechanisms, including unconsciousness, immobility, lack of response to external stimuli, and limited memory upon awakening, as detailed in Figure 1. Anesthetics exert their effects partly by inhibiting wake-promoting neurons or activating sleep-promoting neurons [19]. Imaging studies have shown a correlation between non-rapid eye movement (REM) sleep and anesthesia through the deactivation of the thalamus, leading to cortical inhibition [19]. Anesthetic agents can induce unconsciousness through the thalamus, brain stem, and cerebral cortex [19]. Slow wave activity (SWA) is an electrophysiological measure of sleep propensity or the tendency toward sleep [20]. A decreased SWA rebound following sleep deprivation can be observed with general anesthesia and a slow-wave or isoelectric electroencephalogram (EEG), suggesting that anesthesia may function similarly to non-REM sleep [20]. The depth of anesthesia influences EEG patterns, with specific changes in frequency and amplitude depending on the anesthetic agent and phases of anesthesia. During induction (Phase 1), beta activity initially increases, then decreases as alpha (8-12 Hz) and delta (0.5-4 Hz) waves increase, reflecting a deepening of anesthesia [20]. In Phase 2, alpha and delta waves increase, similar to Stage 3 non-REM sleep, with higher concentrations of agents like propofol and sevoflurane causing a more notable increase in delta waves [20]. In Phase 3, the EEG exhibits burst suppression, marked by alternating bursts of activity, signifying deep CNS depression, typically observed with high doses of propofol or isoflurane [20]. Lower doses of anesthetic agents shift the EEG from beta to alpha and delta activity, while higher doses lead to burst suppression [20]. Data shows that increased sleep pressure, the growing biological urge to sleep from prolonged wakefulness, enhances anesthetic effects [20]. This pressure is mainly driven by the accumulation of adenosine in the brain, which induces tiredness and signals the need for sleep [20]. In contrast, preoperative sleep disturbances and reduced REM sleep are associated with an increased risk of postoperative delirium, particularly in vulnerable populations [20].

Propofol, etomidate, and barbiturates activate GABA-mediated channels and prolong post-synaptic inhibitory currents, suppressing neuronal excitability [19]. Although propofol induces a sleep-like state resembling non-REM sleep, it suppresses REM and slow wave sleep (SWS) [19]. Appropriate propofol administration is essential to minimize its adverse impacts on sleep quality. Strategies such as optimizing dosing, ensuring adequate analgesia, and employing multimodal pain management techniques can help mitigate the adverse effects on sleep architecture [19]. By inhibiting the NMDA receptor, ketamine reduces excitatory signals in neuronal circuits, leading to unconsciousness [19]. The dissociative state induced by ketamine promotes glutamate release from various brain regions, such as the nucleus accumbens, prefrontal cortex, and anterior cingulate [19]. However, volatile anesthetics such as isoflurane and sevoflurane are known to reduce glutamate release and inhibit its uptake [19]. Volatile anesthetics do not substitute natural sleep as most patients experience post-exposure REM sleep rebound [19]. Isoflurane use showed a shift in non-REM sleep from SWS to lighter stages I and II, while isoflurane and sevoflurane inhibit wake-active orexinergic neurons [19]. Wake-active orexinergic neurons in the lateral hypothalamus stabilize sleep-wake cycles by firing more during wakefulness and releasing orexin-A and orexin-B peptides to promote alertness [19]. The neural circuits responsible for awakening from anesthesia differ from those required to induce anesthesia, though they closely resemble the circuits involved in triggering arousal [19]. 

While anesthetics positively affect sleep pathways and the healing benefits of natural sleep on the central nervous system, the immune system also gains advantages. These include a reduced risk of infection and an increased likelihood of survival from sepsis [19]. This potential for immune system enhancement offers a hopeful perspective on using anesthetics. Acknowledging these effects is imperative to providing the best perioperative care and improving surgical patients' recovery.

Tailoring intraoperative anesthesia for optimal sleep experience

Tailoring anesthetic regimens to patients' needs can improve intraoperative care and postoperative recovery [21]. A thorough sleep history and pre-existing sleep disorder assessment can guide the choice and technique of anesthetic agents. Determining pre-existing sleep disorders and age, comorbidities, and medication use can significantly influence a patient's response to anesthesia [21]. Precision medicine and personalized anesthesia are rapidly evolving practices that integrate a patient's genetic and clinical history to enhance patient safety and optimize therapeutic efficacy [21]. By recognizing risks, patterns, and physiological associations, anesthesiologists implement strategies to minimize sedation and the potential disruption of natural sleep patterns [21]. 

OSA is a common diagnosis that is concerning to anesthesiologists due to its increased risk of morbidity and mortality and difficulty in management [22]. A patient with OSA may benefit from regional anesthesia as the route of anesthetic administration. Lower dosages of sedative drugs may also be beneficial for patients with OSA to prevent aggravating airway problems [22]. In regional anesthesia, an anesthetic agent will target a peripheral nerve to block transmission and avoid the onset of pain [23]. Common types of regional anesthesia include spinal anesthesia, epidural anesthesia, peripheral nerve blocks, and intravenous regional anesthesia. A key advantage of regional anesthesia is its ability to reduce the need for general anesthesia during procedures. It does not affect the patient's level of consciousness while providing pain relief and helps maintain normal sleep cycles during recovery [23]. Avoiding general anesthesia offers several benefits, including reduced pain, fewer side effects from systemic drugs, faster recovery, and no need for airway manipulation [23]. Employing techniques that minimize sleep disruptions and address the specific needs of patients with sleep disorders can enhance recovery and improve long-term outcomes. This patient-centered approach ensures optimal perioperative care and highlights the importance of incorporating sleep health into anesthetic planning and execution.

Common postoperative sleep disturbances

Effective management of postoperative sleep disturbances is essential, as they can greatly impact recovery and increase morbidity [24]. Various factors contribute to postoperative sleep issues, including the severity of surgical pain, medication side effects, environmental factors, and disruptions in circadian rhythm [24]. Pain, in particular, is the most common cause of sleep disturbance. It negatively affects sleep quality by inducing anxiety, increasing awakening frequency, and causing discomfort, all of which complicate the recovery process [25].

Opioids play a significant role in postoperative pain management by binding to opioid receptors in the central nervous system, thereby slowing down the transmission of pain signals [25]. They primarily act on mu-opioid receptors, but interactions with delta and kappa receptors also contribute to their analgesic effect [25]. While opioids effectively relieve postoperative pain, it is essential to consider their side effects. Opioids reduce REM sleep and promote awakening and arousal [24]. Studies have established a direct link between sleep and pain, with poor sleep quality leading to increased pain sensitivity and higher analgesic intake [24]. Opioids induce a dose-dependent increase in arousal during sleep-wake periods [26]. They can induce sedation and wakefulness and significantly affect the decline of theta waves, REM sleep, and SWS [26]. 

Opioids can disrupt the body's circadian rhythm, making it difficult to fall or stay asleep, and may lead to fatigue and impaired cognitive function [27]. They can also cause respiratory depression, reducing oxygen saturation and respiratory rate during sleep, which increases the risk of sleep apnea, particularly in patients with pre-existing respiratory conditions [26]. Anesthesia and surgical interventions can also affect the body's circadian rhythm, as the drugs used in general anesthesia primarily target NMDA receptors or act as GABA agonists [27]. The neurons in the suprachiasmatic nucleus contain both NMDA and GABA receptors, and their activation can disrupt the circadian timing system [27]. 

Benefits and strategies for prioritizing sleep in postoperative recovery

The body needs quality sleep to repair and replace cellular components for biological processes, including muscle repair, protein synthesis, tissue growth, and hormone regulation [28]. Postoperatively, patients who achieve restorative sleep exhibit faster tissue healing than those with sleep disturbances, as the body can efficiently control and eliminate inflammation at surgical sites [28]. Cognitive deficits like confusion, memory loss, and difficulties concentrating can result from postoperative sleep problems, making recovery more challenging. By recognizing the critical role of sleep in the recovery process, healthcare providers can implement targeted interventions to promote better sleep quality, ultimately leading to improved outcomes for surgical patients.

Various strategies can be employed to address sleep disturbances, each targeting different aspects of the recovery environment and patient experience. The goal of multimodal pain management is to maximize pain relief while reducing dependency on opioids by combining various analgesics and strategies. Pain pathways can be targeted by medications with multiple mechanisms, resulting in additive and synergistic effects. The most common effective agents are alpha-2 agonists, NMDA receptor antagonists, gabapentinoids, dexamethasone, nonsteroidal anti-inflammatory drugs (NSAIDs), acetaminophen, and duloxetine [29]. The use of non-opioid analgesics, such as acetaminophen and NSAIDs, has been shown to manage pain with fewer side effects [29]. This is particularly important as opioids are known to disrupt sleep architecture by decreasing REM and SWS [25]. Providing information on practices such as establishing a regular sleep schedule, creating a calming pre-sleep routine, and employing relaxation techniques can enable patients to enhance their sleep quality independently. 

Proactively addressing possible sleep-related problems with systematic assessments can strengthen postoperative recovery. This approach enables diagnostic possibilities to be explored during the screening process and facilitates referrals to subspecialists before surgical intervention, helping to prevent complications. Validated tools such as the Pittsburgh Sleep Quality Index (PSQI) standard component of the preoperative workup can facilitate the early identification of sleep disorders in surgical patients [30]. The PSQI is commonly used in clinical practice to assess sleep quality, sleep duration, a variety of sleep disturbances, and a comprehensive overview of a patient's sleep patterns [30].

Future Directions and Recommendations

Studies have shown that preoperative and postoperative sleep disturbances significantly affect the recovery process and the patient's sleep quality after a procedure [7]. The type of anesthetic agents and multimodality pain relief impact the body's physiological function, influencing sleep patterns. While opioids can be beneficial in controlling pain, they can also have a detrimental effect on sleep quality by decreasing REM sleep and heightening arousal, potentially impeding cognitive and mental recovery [27]. However, the potential of non-opioid analgesic regimens and therapies to enhance the quality of sleep and reduce reliance on opioids is a promising development, helping to maintain critical restorative sleep stages crucial for healing. Recent research has demonstrated that anesthetics have a distinct effect on sleep cycles, with inhaled and intravenous types frequently leading to disturbances in REM and SWS [27]. Additional research is necessary to determine how long the potential benefits of Ketamine in maintaining the healing qualities of sleep will last.

Emerging gaps in the literature highlight the importance of long-term studies investigating the lasting impacts of anesthetics on sleep quality and recovery. The potential long-term effects of sleep disturbances on patients' general health, including chronic pain, cognitive decline, and mood problems, should be given further consideration. Patient care can be shaped by thorough research and data analysis on the specific effects of anesthetic drugs in different populations, such as children, the elderly, and individuals with obesity. These populations display distinct physiological reactions that could impact how anesthesia affects sleep after surgery. Creating personalized anesthetic strategies that consider age, body composition, and existing medical conditions is necessary for optimal sleep quality and recovery outcomes. Examining the impact of preoperative interventions such as CBT-I or melatonin supplements could provide strategies to improve postoperative outcomes [14]. 

Healthcare professionals should acknowledge the importance of sleep in perioperative care and promote interventions that enhance sleep quality to strengthen the body's ability to cope with surgical stress and reduce complications. Continued research will be crucial in advancing surgical care through further investigation of the role of sleep in anesthesia.

## Conclusions

This review highlights the critical link between anesthesia and sleep physiology in the perioperative setting. Disruptions to standard sleep patterns from anesthetic agents, comorbid health conditions, and environmental factors can contribute to increased postoperative complications, exacerbated sleep disorders, delayed healing, and heightened pain perception. Improving outcomes and enhancing recovery can be achieved by incorporating the patient's sleep physiology into preoperative planning and postoperative care. Tailoring anesthesia plans and implementing targeted interventions based on individual sleep patterns can optimize the surgical experience, leading to shorter hospital stays and greater patient satisfaction.
